# Silicone Implants with Smooth Surfaces Induce Thinner but Denser Fibrotic Capsules Compared to Those with Textured Surfaces in a Rodent Model

**DOI:** 10.1371/journal.pone.0132131

**Published:** 2015-07-07

**Authors:** Sebastian Fischer, Christoph Hirche, Matthias A. Reichenberger, Jurij Kiefer, Yannick Diehm, Srinivasan Mukundan, Muayyad Alhefzi, Ericka M. Bueno, Ulrich Kneser, Bohdan Pomahac

**Affiliations:** 1 Department of Surgery, Division of Plastic Surgery, Brigham and Women’s Hospital, Harvard Medical School, Boston, Massachusetts, United States of America; 2 Department of Hand-, Plastic and Reconstructive Surgery, BG Clinic Ludwigshafen, University of Heidelberg, Ludwigshafen, Germany; 3 Ethianum, Clinic for Plastic and Reconstructive Surgery, Aesthetic and Preventive Medicine at Heidelberg University Hospital, Heidelberg, Germany; 4 Department of Radiology, Brigham and Women’s Hospital, Harvard Medical School, Boston, Massachusetts, United States of America; University of Salerno, ITALY

## Abstract

**Purpose:**

Capsular contracture is the most frequent long-term complication after implant-based breast reconstruction or augmentation. The aim of this study was to evaluate the impact of implant surface properties on fibrotic capsule formation in an animal model.

**Materials and Methods:**

Twenty-four rats received 1 scaled down silicone implant each; 12 of the rats received implants with textured surfaces, and the other 12 received implants with smooth surfaces. After 60 and 120 days, rats in each group underwent 7-Tesla Magnetic Resonance Imaging (MRI) and high-resolution ultrasound (HR-US), and specimens of the capsules were acquired and used to measure capsule thickness through histology, collagen density through picro sirius red staining, and analyses of expression of pro-fibrotic and inflammatory genes (Collagen1-4, TGFb1, TGFb3, Smad3, IL4, IL10, IL13, CD68) through qRT-PCR. Furthermore, MRI data were processed to obtain capsule volume and implant surface area.

**Results:**

On day 60, histology and HR-US showed that fibrotic capsules were significantly thicker in the textured implant group with respect to the smooth implant group (p<0.05). However, this difference did not persist on day 120 (p=0.56). Capsule thickness decreased significantly over the study period in both smooth and textured implant groups (p<0.05). Thickness measurements were substantiated by MRI analysis and volumes changed accordingly. Implant surface area did not vary between study dates, but it was different between implant types. On day 60, the density of collagen in the fibrotic capsules was significantly lower in the textured implant group with respect to the smooth group (p<0.05), but again this difference did not persist on day 120 (p=0.67). Collagen 1 and CD68 were respectively over- and under expressed in the textured implant group on day 60. Significant differences in the expression of other genes were not observed.

**Conclusion:**

Silicone implants with textured surfaces led to temporarily thicker but less dense fibrotic capsules compared with smooth surfaces. 7-Tesla MRI and HR-US are capable for non-invasive in-vivo assessment of capsular fibrosis in an animal model and can provide unique insights into the fibrotic process by 3D reconstruction and surface area measurement.

## Introduction

According to the American society of plastic surgeons, about 400,000 women in the United States only undergo cosmetic or reconstructive breast augmentations annually, making insertion of silicone implants the most frequently performed intervention in plastic surgery [1]. Although no other biomaterial reveals comparable properties in terms of availability, adaptability and immunogenicity, silicone remains a foreign body that is prone to a physiological and obligatory foreign body reaction [2]. Thereby, the inserted silicone gets encapsulated by fibrotic tissue, which in spite of intending to protect the organism against potentially harmful materials, can actually lead to a harmful complication itself—namely capsular contracture [3]. Visible deformities, palpable hardness and progressive pain make capsular contracture clinically relevant in up to 30% of cosmetic and even 73% of reconstructive cases after radiation therapy, and thus capsular contracture is the most common long-term complication after silicone breast reconstruction and augmentation [4–6]. Proposed mechanisms include direct immunostimulation and sub-clinical infection, which are held mainly responsible for the initiation and maintenance of capsule formation [7, 8]. Both mechanisms are capable of inducing a chronic inflammatory reaction that stimulates proliferation and differentiation of fibroblasts and the ensuing synthesis of collagen and other extracellular matrix proteins. Whereas sterile working conditions and perioperative antibiotics reduce the risk of infection, modifications of the silicone implant, especially its surface, were invented to increase biocompatibility and thus decrease capsular fibrosis [9–11]. Today, smooth, textured and poly-urethane covered implants are available. Although the latter revealed good results in recent studies, removal can be painstaking when necessary, and foremost within the context of the ongoing discussion about cancer induction, poly-urethane covered implants are the less preferred therapeutic option for most surgeons [4, 12]. In contrast smooth or textured implants are commonly used in clinical practice for breast augmentation. Interestingly, the incidence of capsular fibrosis varies significantly in current literature and the choice of surface type relies mainly on the personal preference of the surgeon. However, in a recently published meta-analysis by Liu et al. only surface properties and in particular smooth surfaces are more likely associated with capsular contracture [13]. Reports such as this demonstrate the impact of the implant’s surface on capsular fibrosis and the need for further studies that can explain these clinical findings. In addition, animal models lack in non-invasive in-vivo assessments of capsule formation, and especially objective ways to assess contracture, which is mandatory for investigation of therapeutic applications in future studies.

Therefore, the aims of this study were to demonstrate the impact of smooth vs. textured surfaces of silicone implants on capsule formation and to verify histological and biochemical findings by non-invasive in-vivo measurements.

## Materials and Methods

### Animal Model

**Fig 1:**
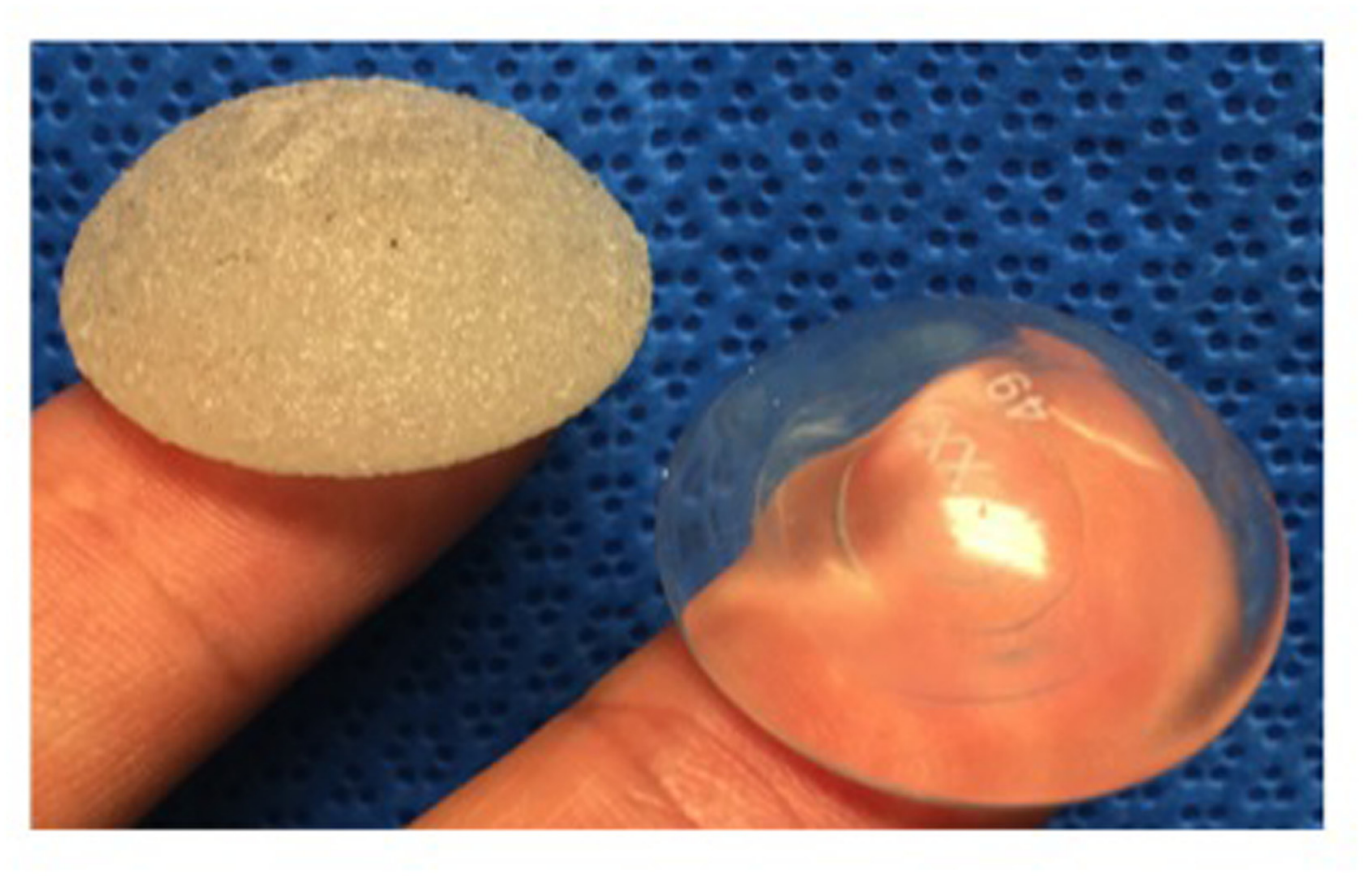
Gel-filled textured (left) and smooth (right) silicone implants (Polytech Health and Aesthetic, Dieburg, Germany).

After approval of the animal experimental protocol (AEP) by the IACUC committee of the Harvard Medical Area Standing Committee on Animals (AEP No.: 05086), twenty-four female Lewis rats (200-225gr./10-12wks.) were randomly divided into four groups of 6 rats each. Two groups (n = 12) received textured (t) silicone implants (Polytech Health and Aesthetic, Dieburg, Germany, gel filled, pore size range of 50–900 μm, Fig 1 left) and two groups received smooth (s) silicone implants (Polytech Health and Aesthetic, Dieburg, Germany, gel filled, 2cm in diameter, Fig 1 right). One sub-group (n = 6) within each implant type was euthanized at 60 days, and the second was euthanized at 120 days after insertion of the silicone device. According to Sengupta et al., one month of an adult rat’s life equates to 3 human years, providing a total follow-up period of approximately 6 and 12 human years, respectively, which is relevant to the human population suffering from capsule contracture [14]. Insertion was performed as described before under sterile conditions through a 4 cm longitudinal paravertebral incision [15]. The implant was placed on the opposite side of the spine above the scapula to prevent interference of capsule formation with scar tissue that might develop from the incision. During implantation particular attention was paid to hemostasis to avoid hematoma formation. Absorbable 4–0 Vicryl sutures were used for wound closure.

Explantation was performed at the given time point by resecting the implant with surrounding capsule en-bloc. For further analysis we utilized the parietal part of the capsule between the implant and the skin.

### Histology

After formalin fixation and paraffin embedding, hematoxylin eosin staining was utilized for capsule thickness assessment. The thickness of the capsules was measured between the cutis and the next tissue that does not belong to the capsule, namely fat or muscle. Measurements were performed with ImageJ (v. 1.46, NIH, USA) at the thickest part of the capsule. To assess collagen density we performed picrosirius red staining and measured pixel density with means of ImageJ (v. 1.46, NIH, USA) in two fields per section randomly chosen by two blinded investigators. All measurements were performed by two blinded observers and expressed as the mean ± standard deviation (SD).

### Magnetic Resonance Imaging

**Fig 2:**
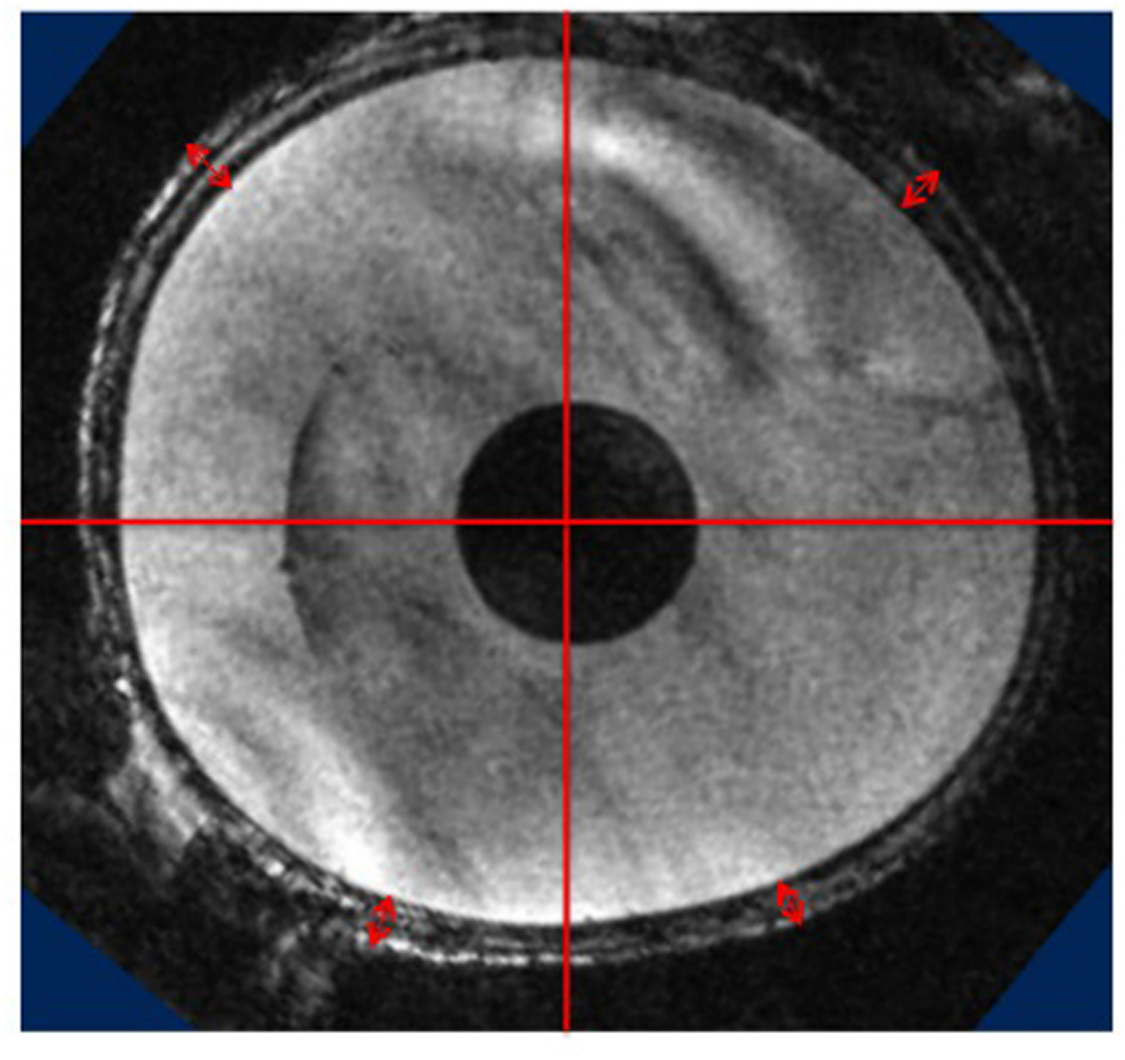
Example picture of MR image in axial plane utilized for thickness measurements. Measurements were performed in each quadrant from the shell of the implant to the outer side of the skin (red arrows). Scale was set with means of the inner circle that had the standardized diameter of 0.8 centimeter.

Magnetic resonance imaging (MRI) was performed on a commercially available UltraHigh Field MRI preclinical scanner, 7.0T Bruker BioSpec (Billerica, MA, USA). Imaging was performed on 2 rats of each study group. Scans were run in the FLASH 3D mode using a volume transmit and receive coil with FOV of 30×30 mm^2^, TR/TE of 15/4.662 ms and a matrix size of 180×240×240. Isotropic spatial resolution of 0.150×0.150×0.150 mm/pixel was achieved in order to allow for accurate measurement in any geometric plane. The imaging parameters were kept identical for each animal and study group. Data were converted to DICOM format and analyzed via ImageJ (v. 1.46, NIH, USA) and 3D Volume measurement plug-in. Thickness measurements were performed in the axial plane, which was perpendicular to the fat-water-shift direction. The most visceral height of this plane was chosen and measurements were made at thickest part of each quadrant and from the outer margin of the implant to the outer side of the skin (Fig 2, red arrows).

**Fig 3:**
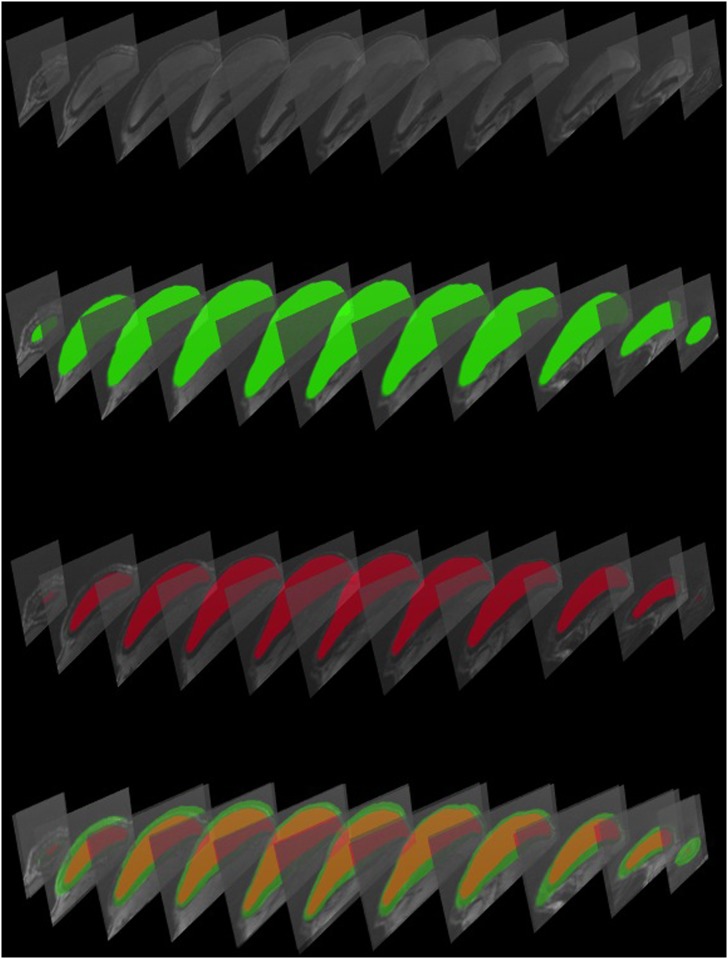
Example pictures of MR images for volumetric measurements. From top to bottom: Blank slides, complete capsule volume (green), implant volume (red) and complete minus implant volume (green margin). Volume calculations were performed via VOLUMEST and the scale was adjusted automatically by the software.

Volumetric measurements were made by VOLUMEST [16] plug-in for ImageJ (v. 1.46, NIH, USA). To assess the Volume of the capsule we subtracted the implant volume from the total capsule volume (Fig 3). To calculate surface area of the implant (A) we used the following formula:

A = (SAE/2) + π * x * y

where SAE is the Knut Thomsen formula for calculation of the surface area of an ellipsoid [17]: SAE = 4π * (((z*x)_1,6075_+(z*y)_1,6075_+(x*y)_1,6075_)/3) _1/1,6075_

π = 3.14159

**Fig 4:**
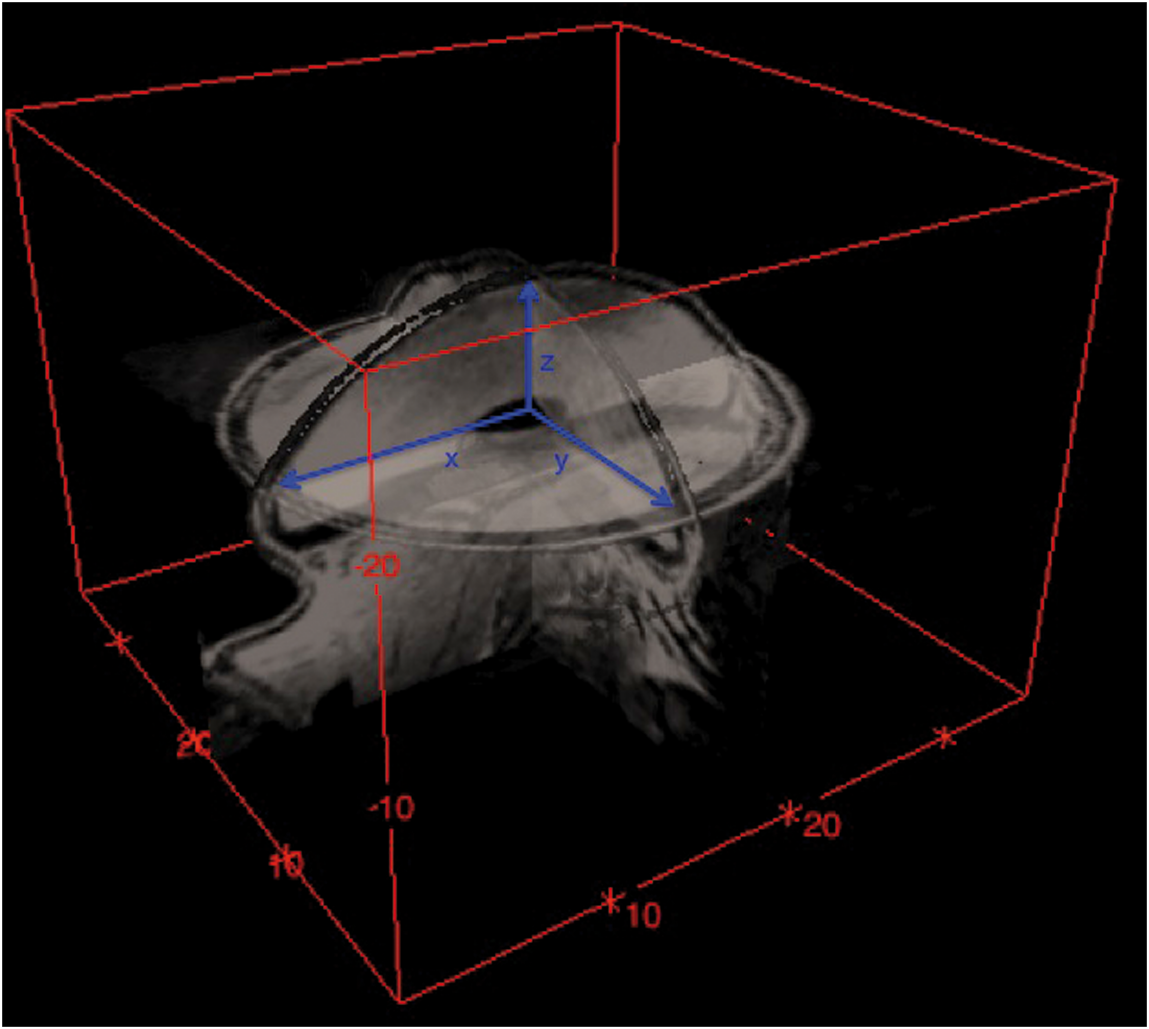
Example picture of MR image after 3D reconstruction and displayed as multi-orthoslice. Measurements were performed at the x-, y- and z-axis (blue arrows). Scale was adjusted automatically by the software.

X, y and z variables were measured from 3D reconstructed MR images with means of 3D viewer tool of ImageJ (v. 1.46, NIH, USA) (Fig 4). By dividing SAE by 2 and adding the surface area of the base of the implant, which is in the shape of an ellipse, we obtain an approximate surface area of the entire implant.

All measurements were performed by two blinded observers and expressed as the mean ± standard deviation (SD).

### High-Resolution Ultrasound

**Fig 5:**
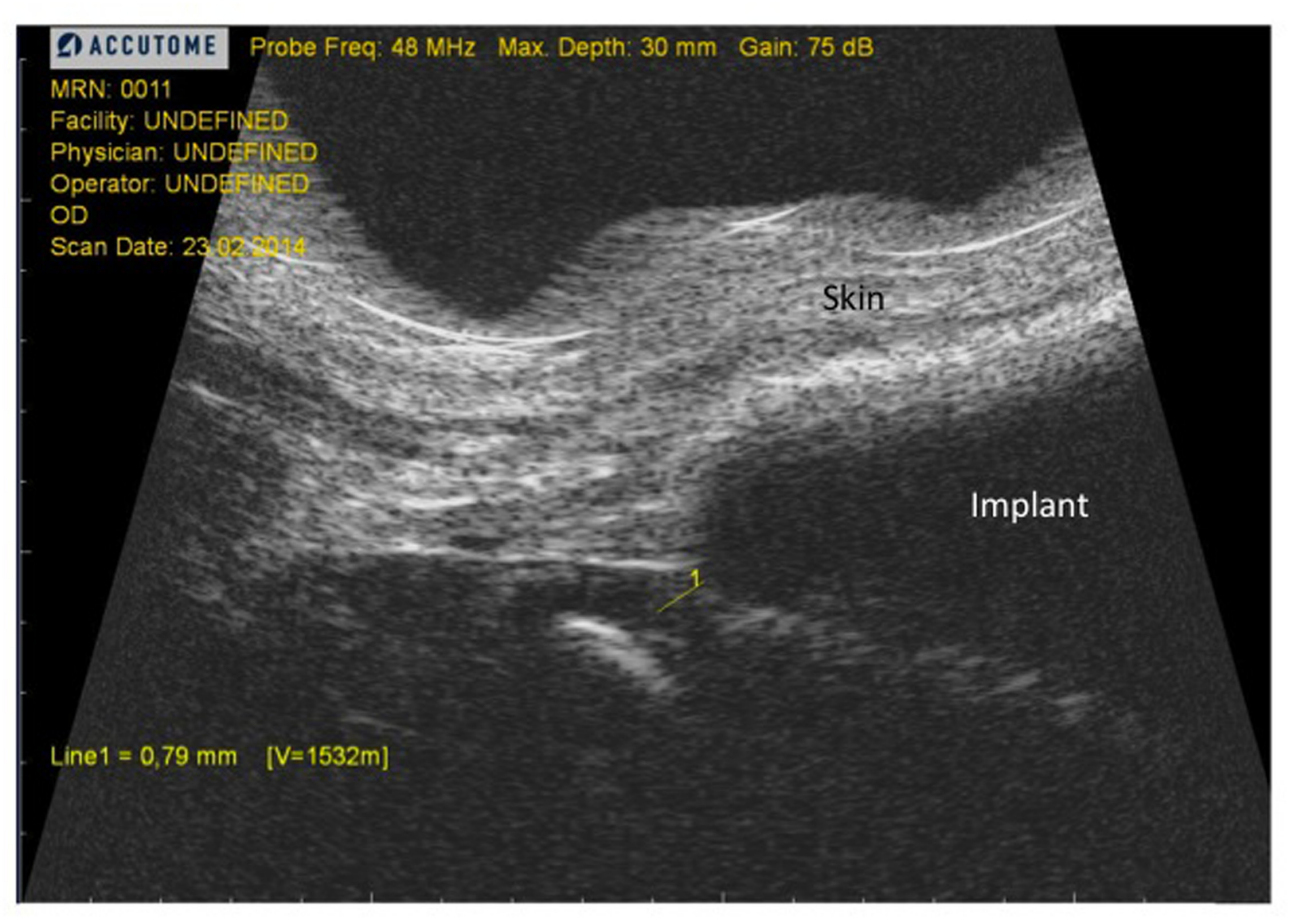
Example picture of HR-US image. Measurements were performed at the outer margin of the implant (yellow line). Scale was adjusted automatically by the software.

High-resolution ultrasound (HR-US) was performed in each rat in a blinded fashion with means of a 48 MHz transducer (Accutome UBM plus, Accutome, Malvern, PA, USA) on day 60 and 120 after insertion, depending on the study group. Conventional transmission gel (Aquasonic 100, Parker Laboratories, Fairfield, NJ, USA) was applied to the shaved skin above the implant and a short video sequence was taken longitudinal across the implant and parallel to the spine. Measurements were performed on the height of the outer margin of the implant (Fig 5, yellow line). This measurement site equates to the measurement site of the MRI. All measurements were performed by two blinded observers and expressed as the mean ± standard deviation (SD).

### Real-Time Quantitative PCR

Total cellular RNA was extracted using RNeasy Mini Kit (Qiagen, Valencia, CA, USA) and reverse transcribed into cDNA by the ABI PRISM TaqMan reverse transcription method. Expression for genes of interest was analyzed in previously shock frozen tissue samples from capsules that were harvested at 60 or 120 days, depending on the study group. Primers were purchased from Life Technologies Corporation (Carlsbad, CA, USA) and are listed in Table 1. Quantitative real-time PCR was performed via ABI PRISM 7900HT Sequence Detection System (Applied Biosystems, Carlsbad, CA) and data analyzed by ExpressionSuite Software v 1.0.4 (Life Technologies Corporation, Carlsbad, CA, USA). This software utilizes the comparative CT (ΔΔ CT) method to quantify relative gene expression. After normalization to the expression of beta-2 microglobulin acting as housekeeping gene, relative gene expression among study groups are given in relative quantitation (RQ).

**Table 1:** List of Primers utilized for TaqMan quantitative real time PCR. Primer names and catalog numbers are presented according to the vendor Life Technologies Corporation (Carlsbad, CA, USA).

Primer name	Catalog numbers
Col1a1	Rn01463848_m1
Col2a1	Rn01533081_m1
Col3a1	Rn01437660_g1
Col4a1	Rn01482927_m1
TGFb1	Rn00572010_m1
TGFb3	Rn01517871_m1
Smad3	Rn01422009_m1
CD68	Rn01495634_g1
IL4	Rn01456866_m1
IL10	Rn00563409_m1
IL13	Rn00587615_m1
B2m (housekeeping gene)	Rn00560865_m1

### Statistical Analysis

The independent samples t-Test was utilized to analyze data related to capsular thickness and volume measurements, implant surface area measurements, collagen density and differences in expression of tested genes. Statistical significance level was set as p<0.05.

## Results

### Histology

#### Capsule thickness

**Fig 6:**
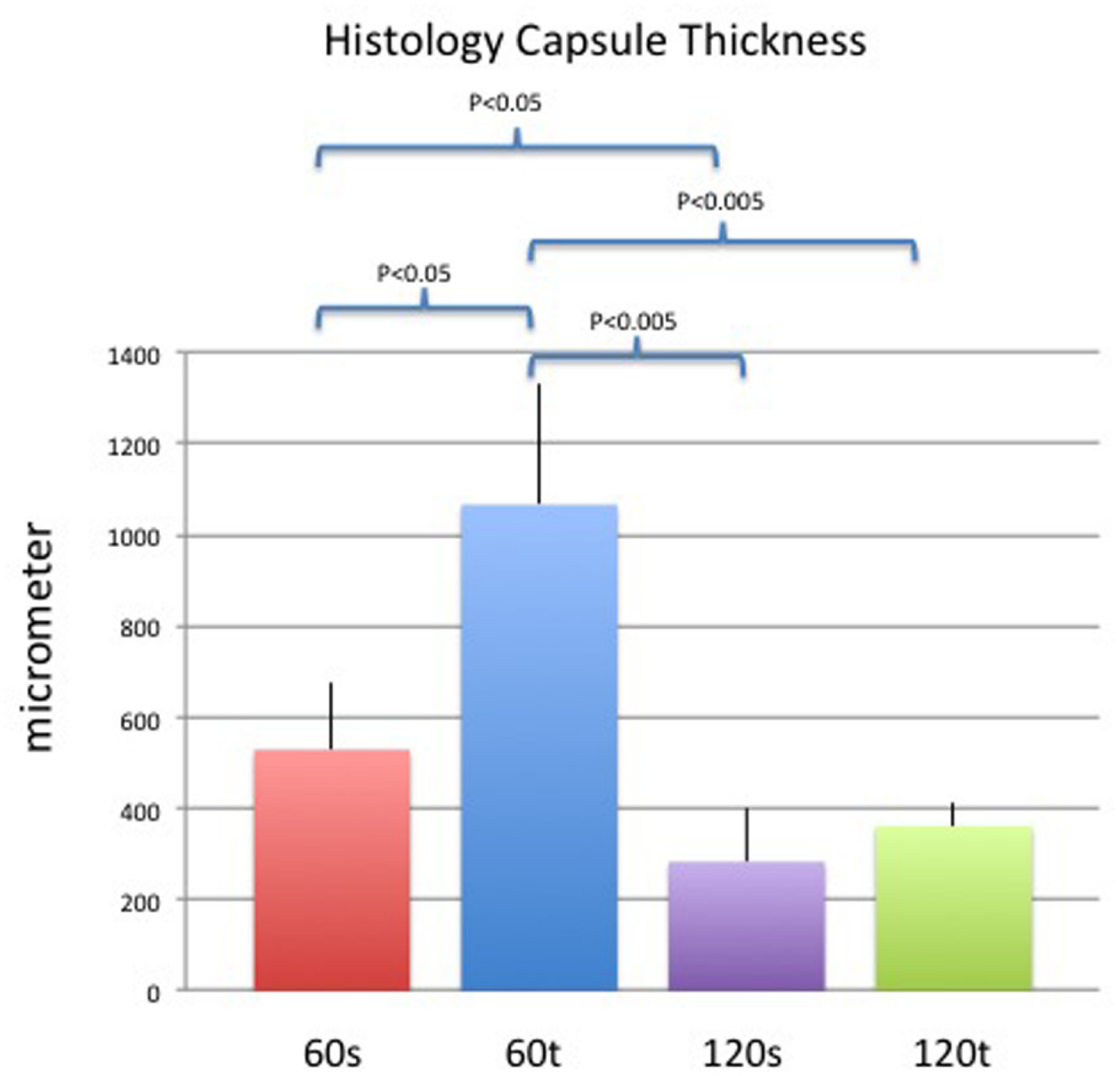
Mean (+/- standard deviation) capsule thickness measurements in histology sections of each study group. P<0.05 and p<0.005 were considered statistically significant and highly significant.

The thickness of fibrotic capsules was significantly different between smooth and textured implants on day 60 (60s: 520.3±149μm vs. 60t: 1066.1±263.9μm; p<0.05) but not on day 120 (120s: 282.9±118μm vs. 120t: 361.6±52.4μm; p = 0.56). Sixty days after insertion of both smooth or textured implants, capsule thickness decreased significantly over time (60s vs. 120s; p<0.05 and 60t vs. 120t; p<0.005). Results are demonstrated in Fig 6.

#### Collagen Density

**Fig 7:**
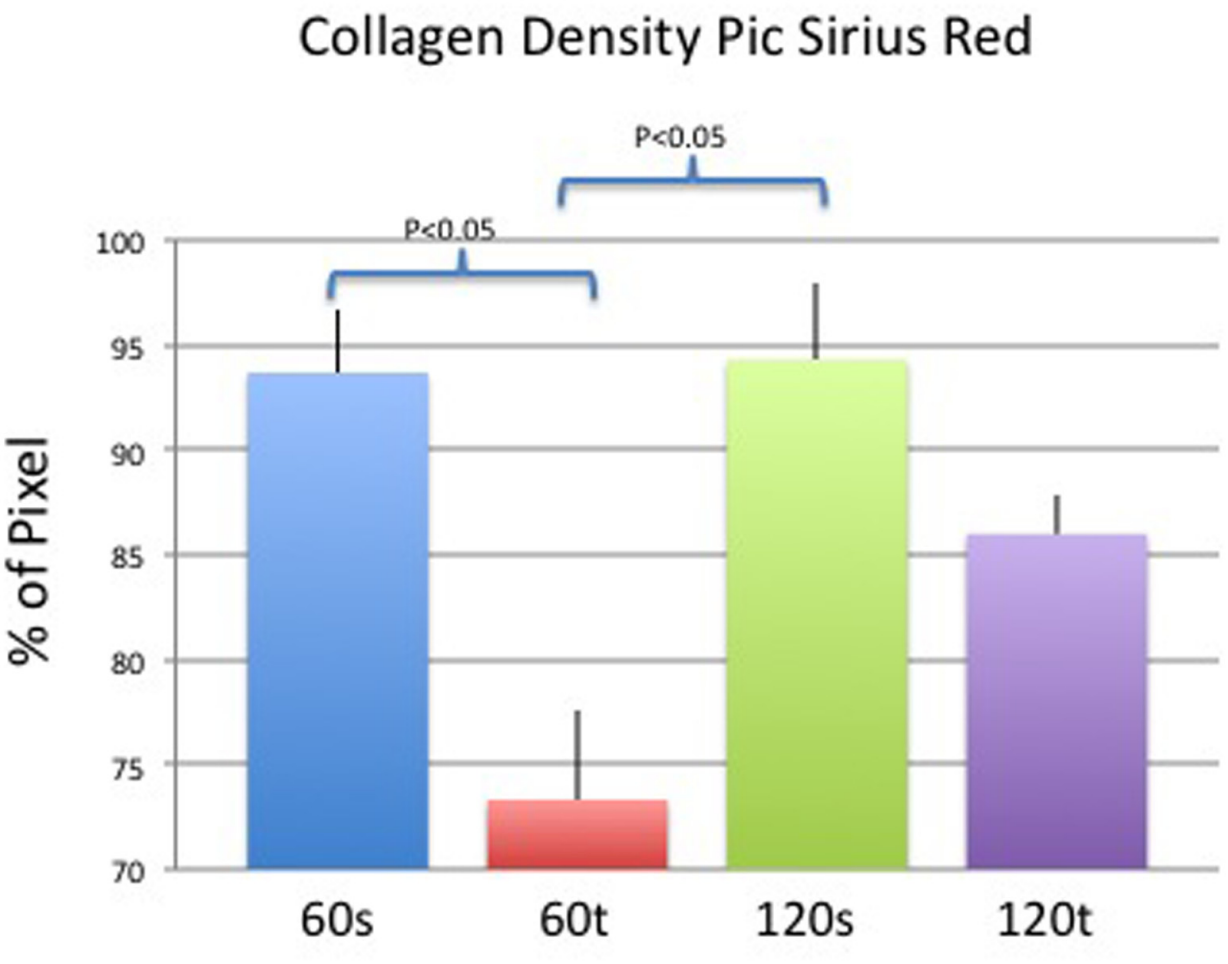
Mean (+/- standard deviation) collagen density in each study group. P<0.05 was considered statistically significant.

**Fig 8:**
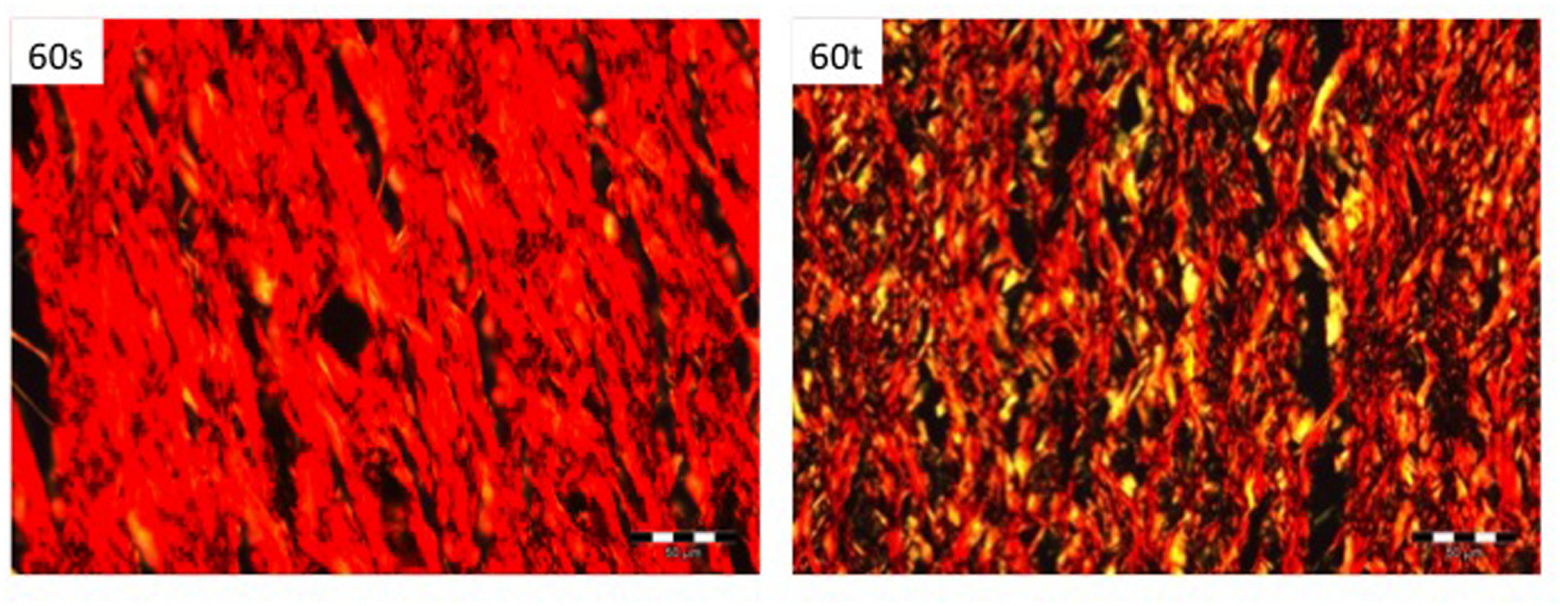
Example pictures of picrosirius red staining under polarized light of both study groups on day 60.

Pixel density varied significantly between smooth and textured implants groups on day 60 (93.6±2.9% vs. 73.3±4.3%; p<0.05). In addition, significant differences were seen between the textured group on day 60 and the smooth group on day 120 (94.3±3.6% vs. 73.3±4.3%; p<0.05). On day 120, we did not observe significant differences in collagen density related to implant surfaces (94.3±3.6% vs. 86±1.8%; p = 0.67). Results are depicted in Fig 7 and examples of histological pictures in Fig 8.

### Magnetic Resonance Imaging

**Fig 9:**
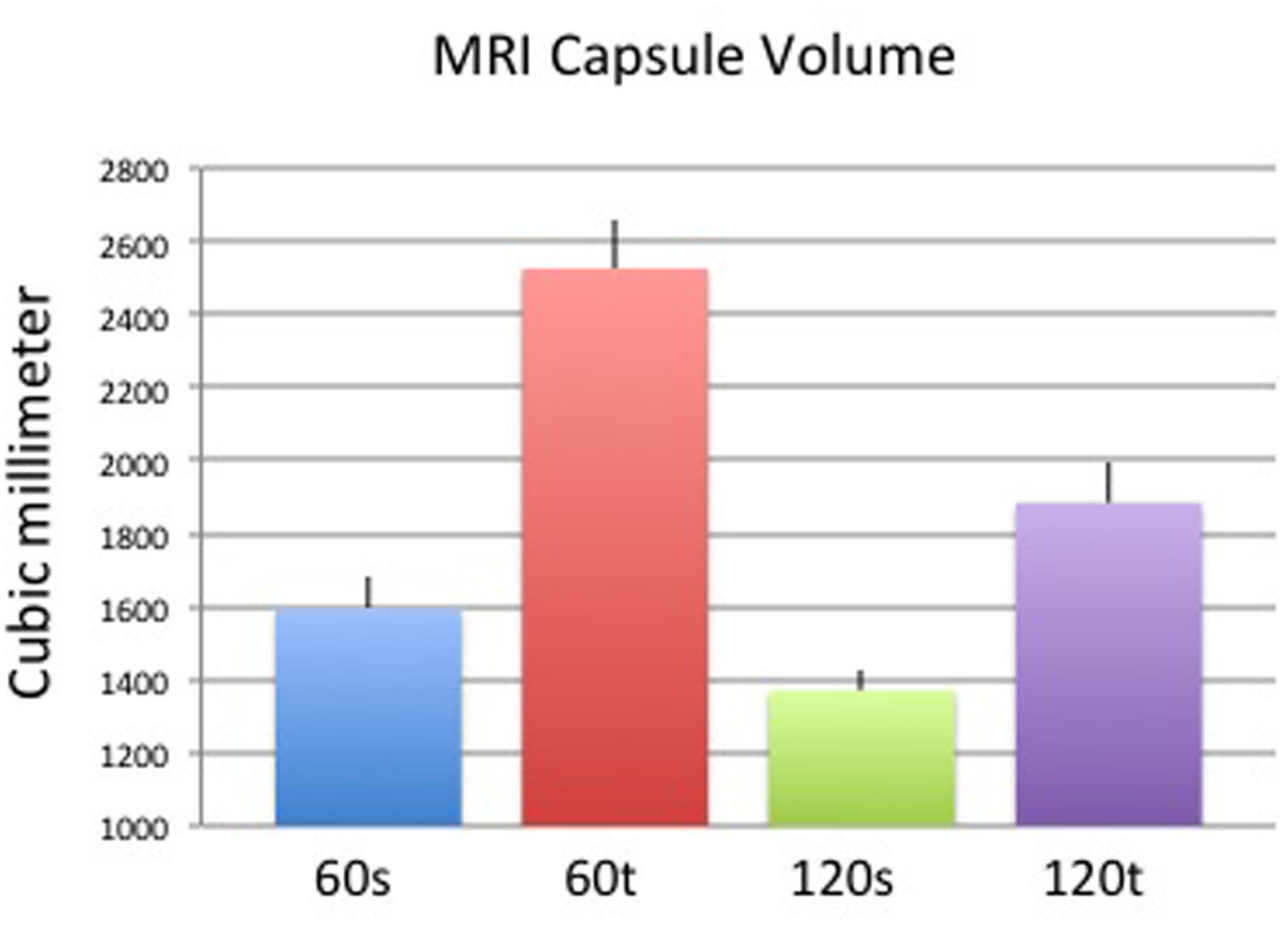
Mean (+/- standard deviation) capsule volume in MRI of each study group. Results were not statistically significant.

**Fig 10:**
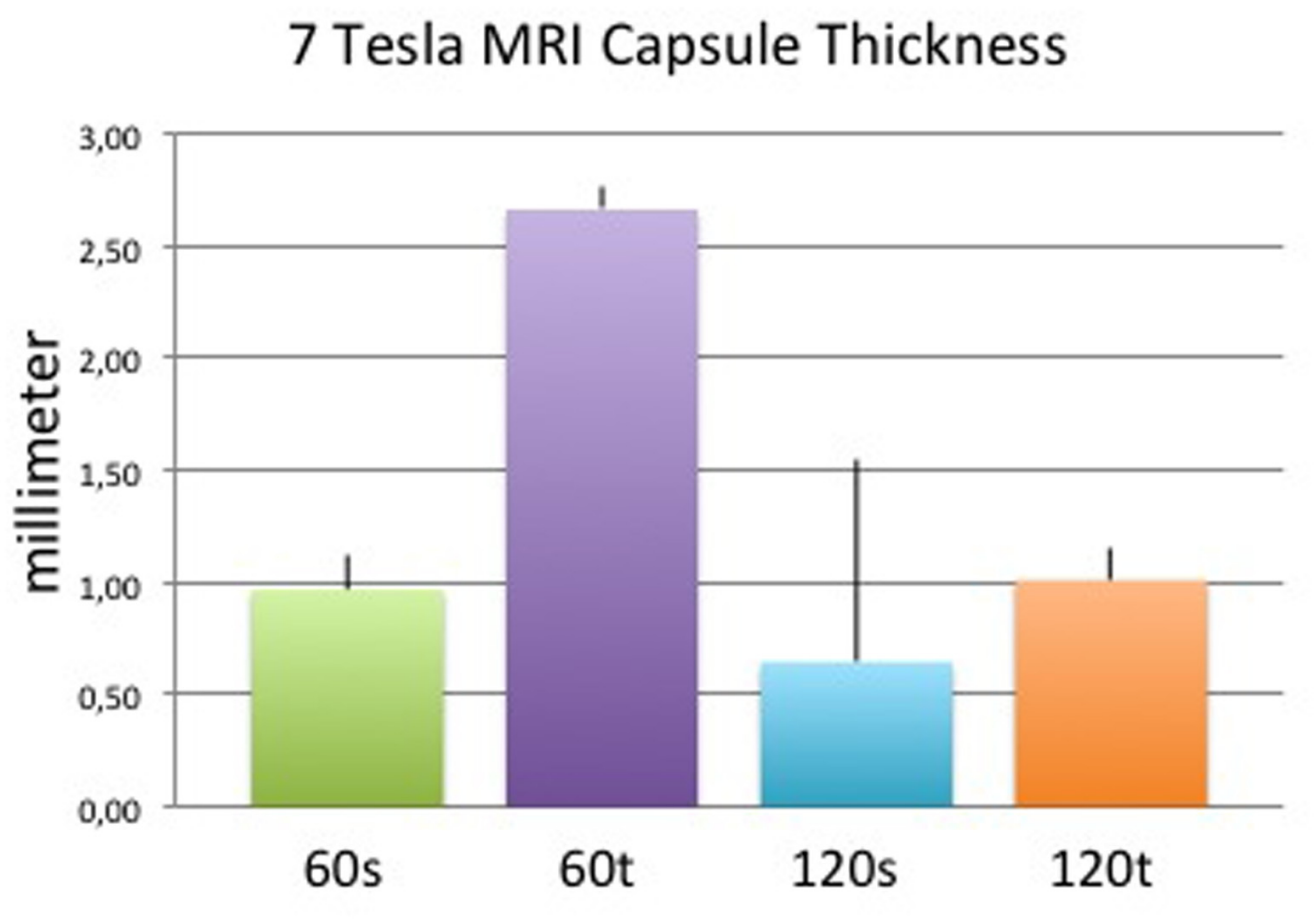
Mean (+/- standard deviation) capsule thickness in MRI of each study group. Results were not statistically significant.

**Fig 11:**
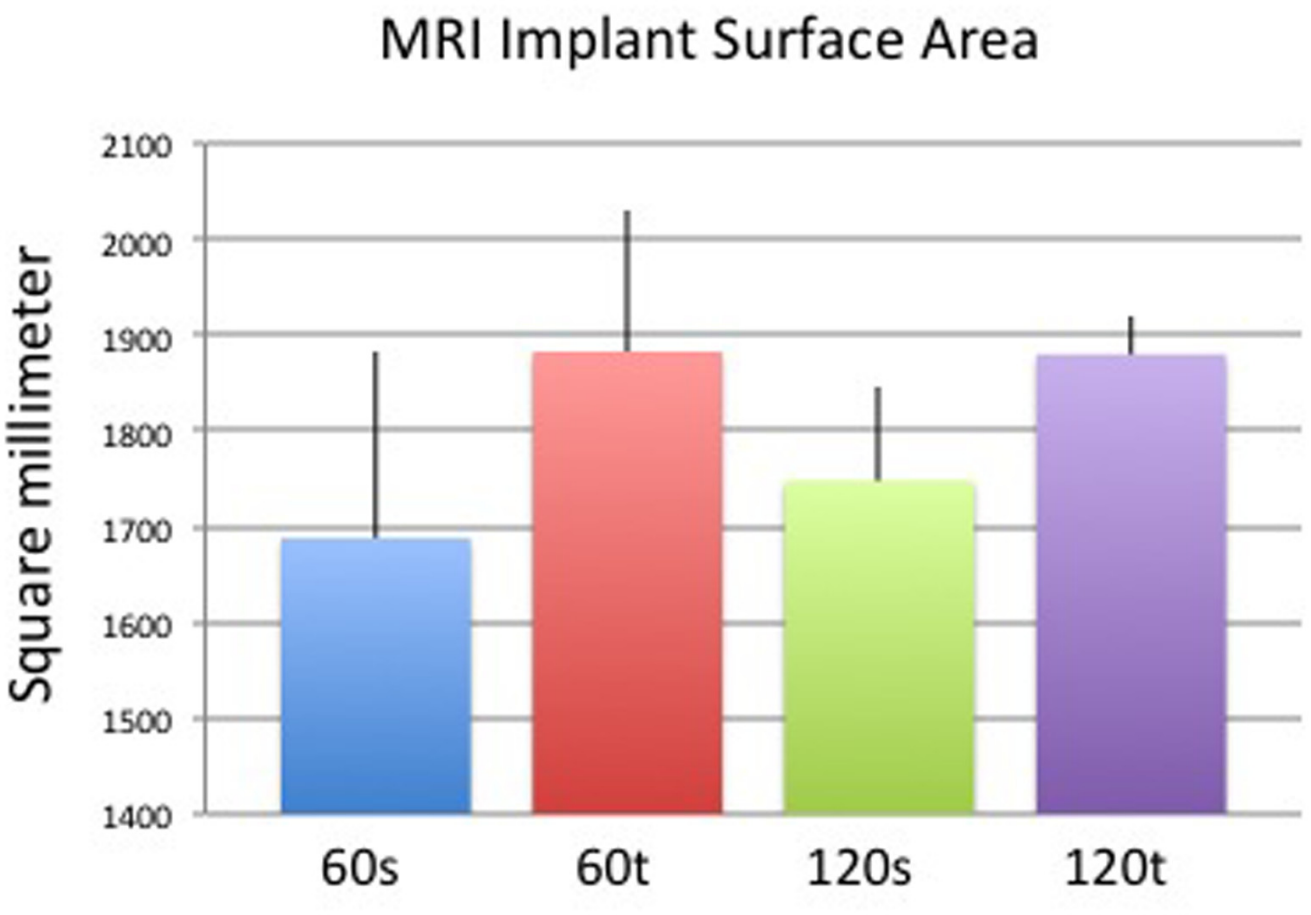
Mean (+/- standard deviation) implant surface area in MRI of each study group. Results were not statistically significant.

**Fig 12:**
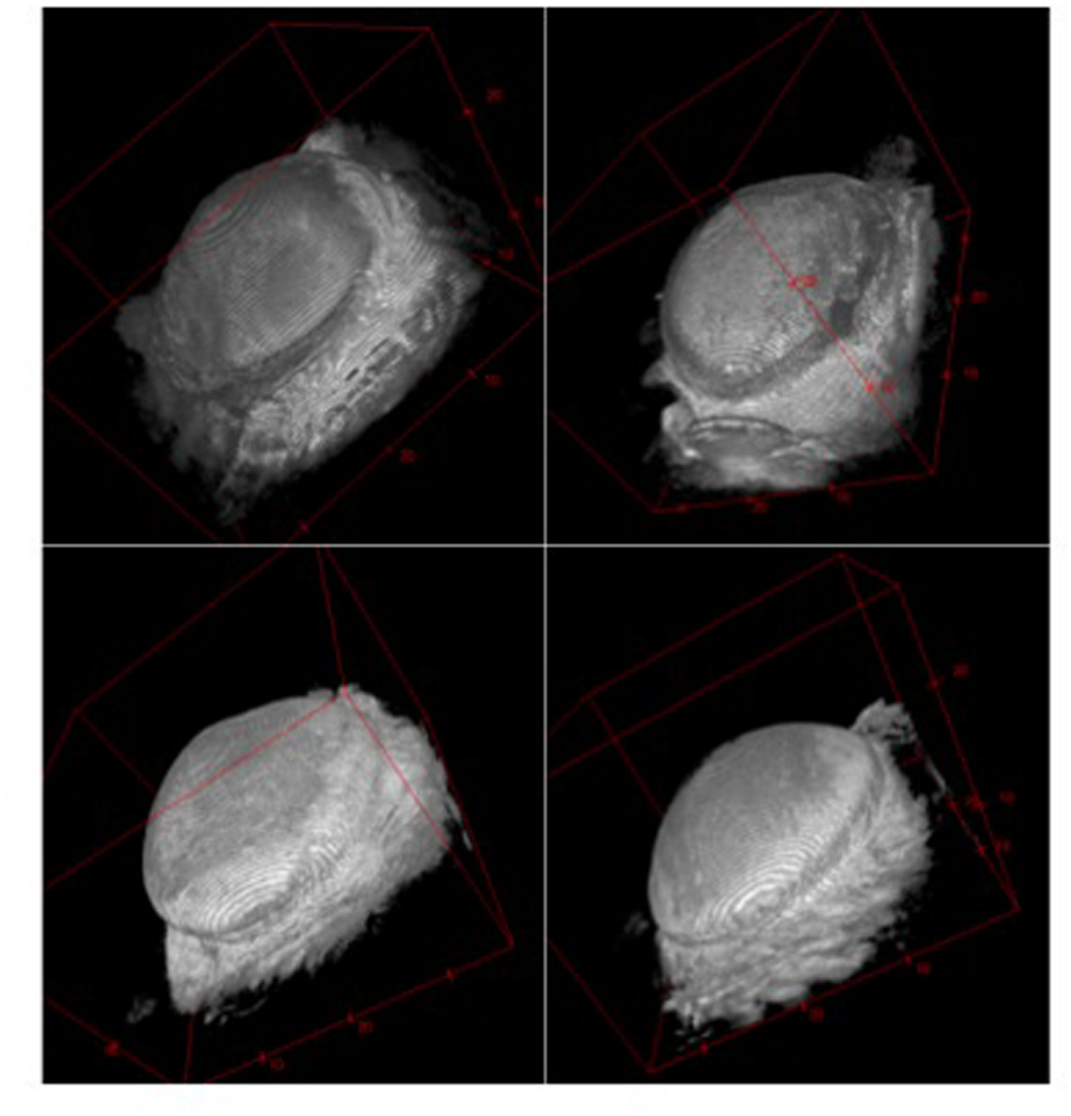
3D reconstruction of 7-Tesla MRI scans of smooth implants on day 60 (60s, upper left), textured implants on day 60 (60t, upper right), smooth implants on day 120 (120s, lower left) and textured implants on day 120 (120t, lower right).

Capsules on day 60 appeared thicker and less dense when surrounding textured implants compared with those surrounding smooth implants, whereas apparent differences elapsed on day 120. Concomitant to thickness measurements, capsule volumes were higher in the textured (vs. smooth) implant group on day 60 and comparable across both surface groups on day 120 (Fig 9). Although the trend of each group correlated with histological and ultrasound measurements, differences in MRI were not significant between groups (Fig 10). Surface areas of implants were higher in the textured group compared to smooth surfaces after both 60 and 120 days. Within the same implant group, surface areas were comparable (Fig 11). Again, MRI-based measurements were not statistically significant between groups. Examples of 3D volume view images are demonstrated in Fig 12.

### High-Resolution Ultrasound

**Fig 13:**
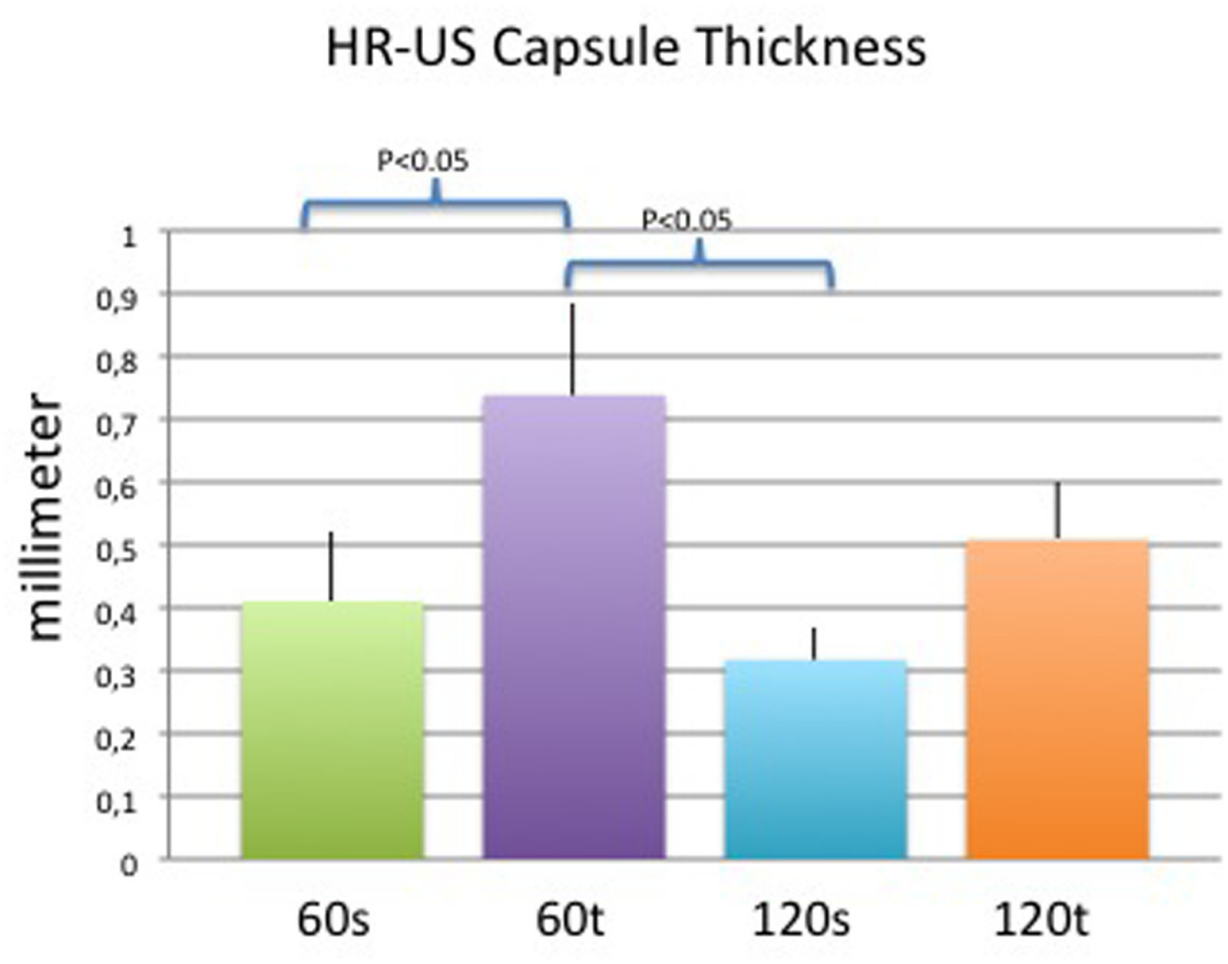
Mean (+/- standard deviation) capsule thickness measurements via high-resolution ultrasound of each study group. P<0.05 was considered statistically significant.

Ultrasound measurements revealed significant differences in thickness of smooth vs. textured implants on day 60 (0.41±0.12mm vs. 0.73±0.12mm; p<0.05) and between textured implants on day 60 and smooth implants on day 120 (0.73±0.12mm vs. 0.31±0.3mm; p<0.05). On day 120, no significant differences related to implant surfaces were found (0.31±0.2mm vs. 0.51±0.1%; p = 0.07). Results are depicted in Fig 13.

### Real-Time Quantitative PCR

**Fig 14:**
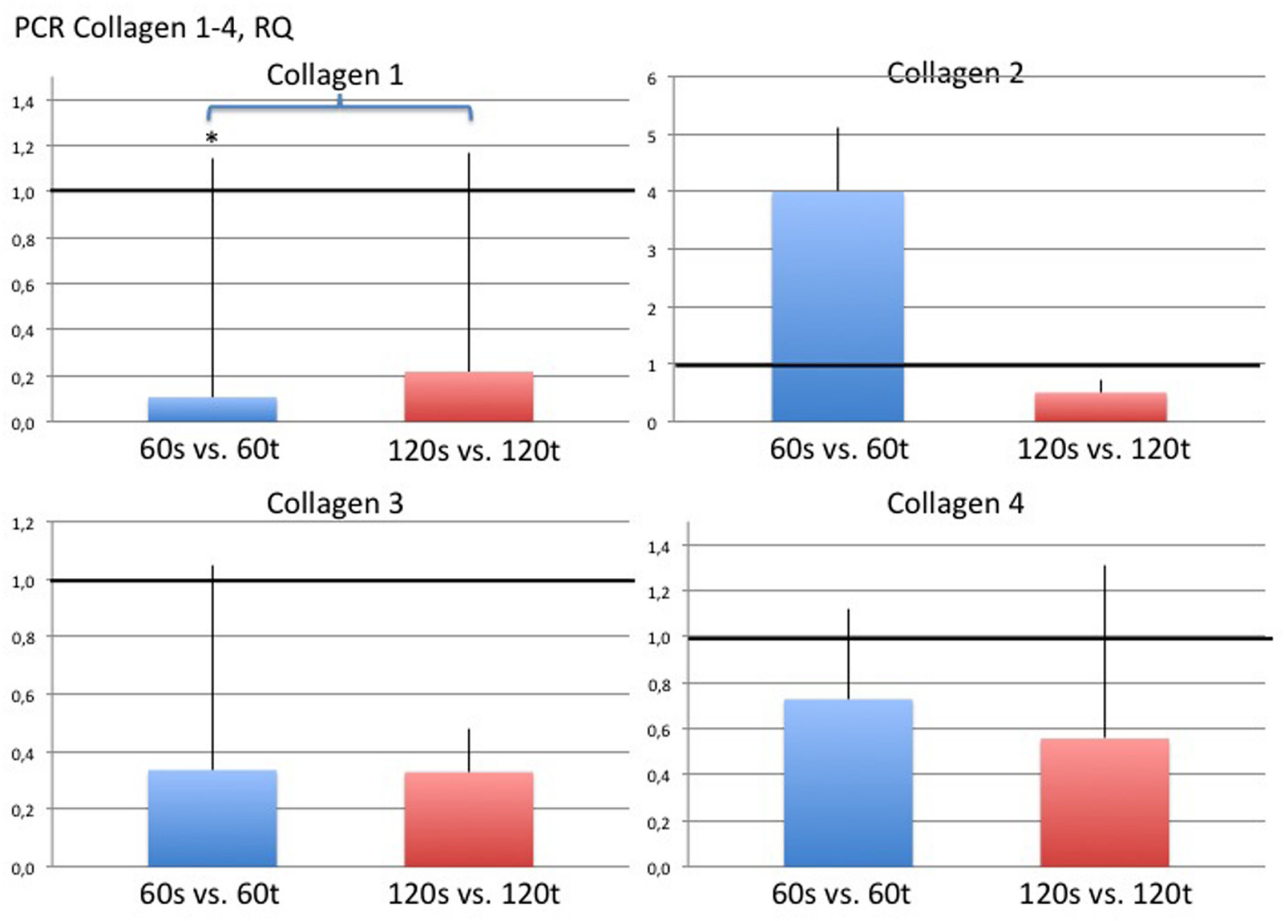
Results of quantitative real time PCR for Collagen 1 (upper left), 2 (upper right), 3 (lower left) and 4 (lower right) given in fold change compared to textured implant group of day 60 or 120, respectively. Asterisks and brackets indicate statistical significance and P<0.05 was considered statistically significant.

**Fig 15:**
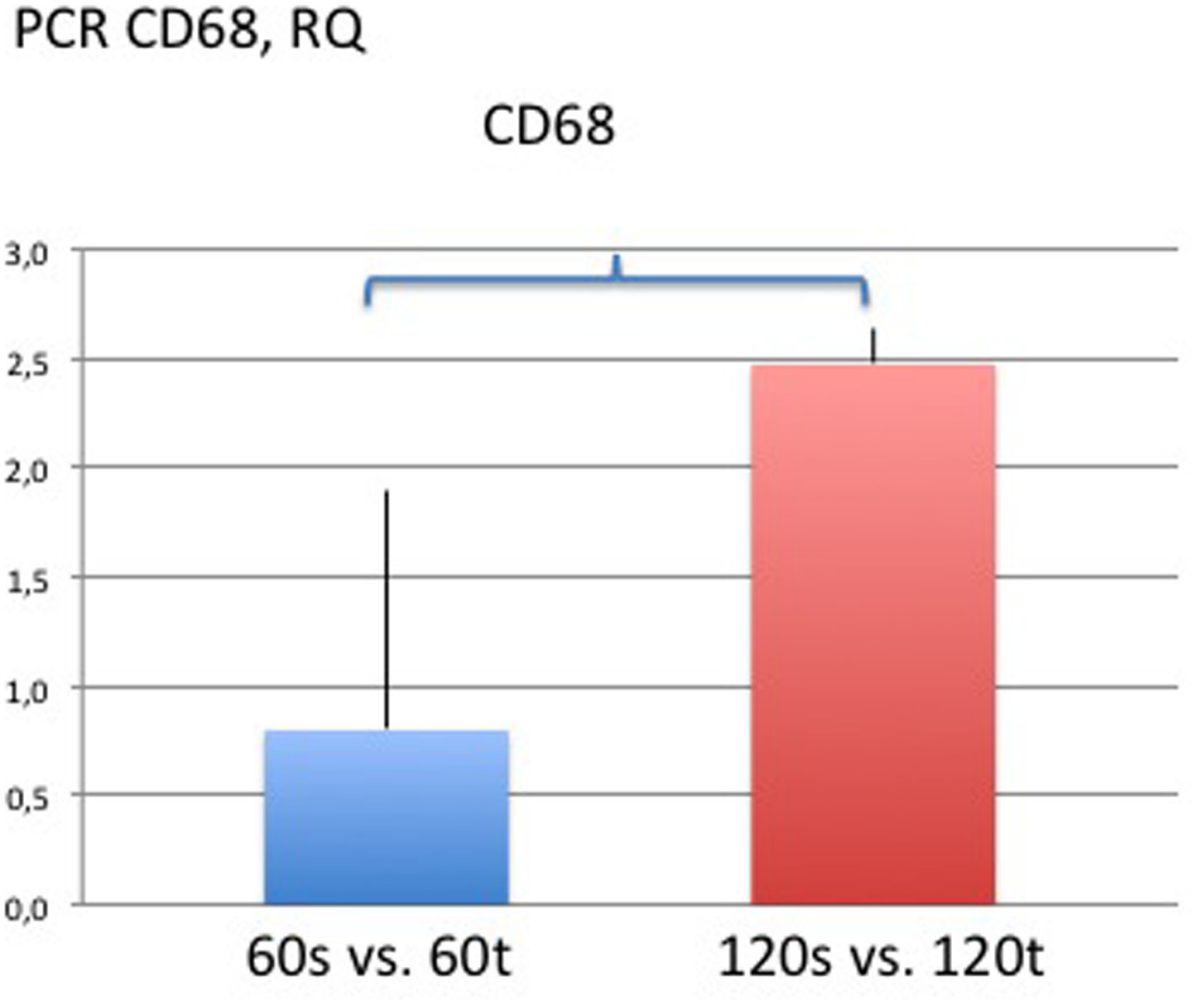
Results of quantitative real time PCR for CD68 given in fold change compared to textured implant group of day 60 or 120, respectively. Asterisks and brackets indicate statistical significance and P<0.05 was considered statistically significant.

**Fig 16:**
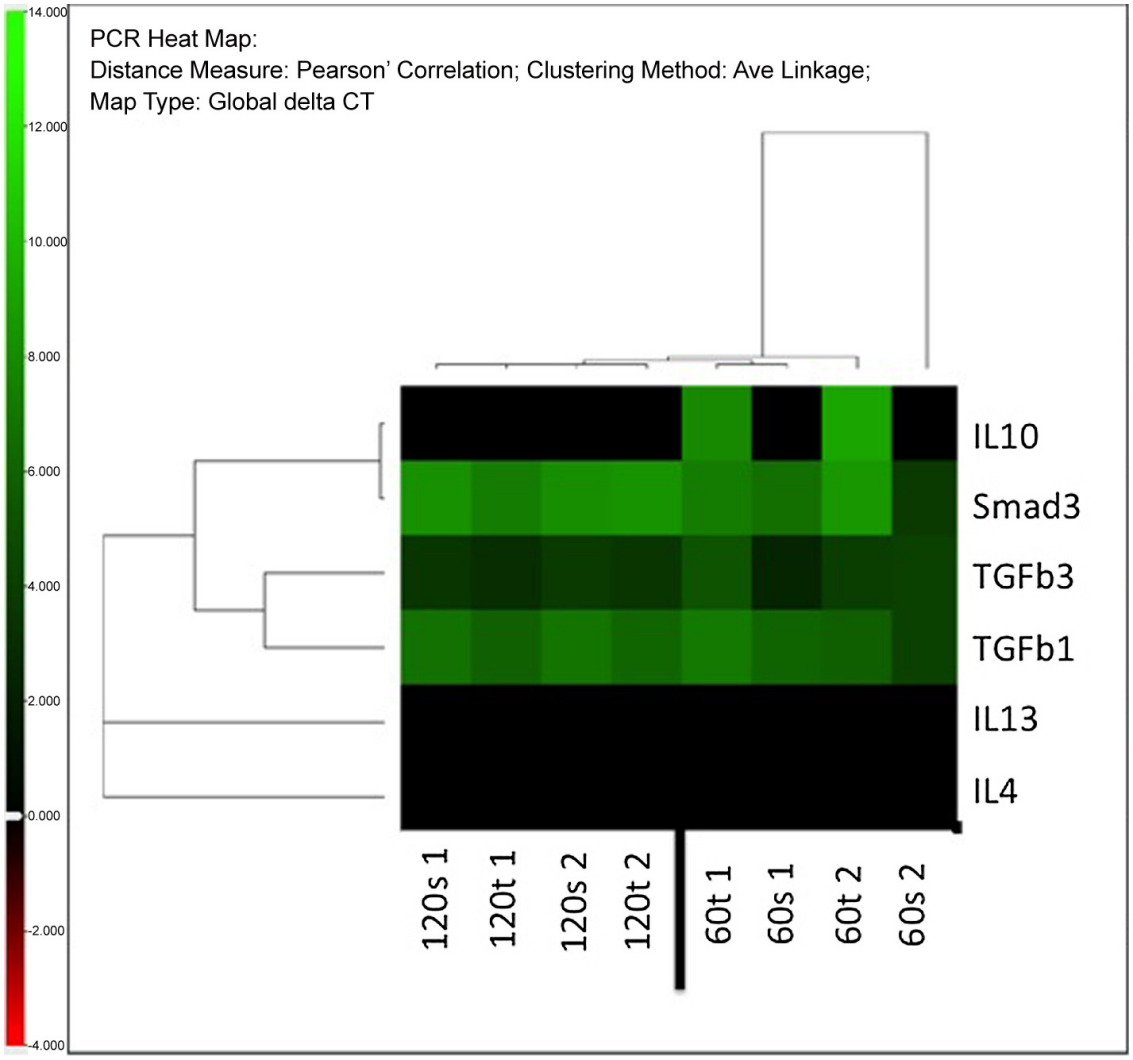
Results of quantitative real time PCR for IL10, IL4, IL13, TGFb-1, TGFb-3 and Smad3 given in delta Ct (Ct gene—Ct housekeeping gene) for each study group and illustrated as heat map (Distance measure: Pearson’s Correlation; Clustering Method: Ave Linkage; Map Type: Global delta CT; Ct > 40,000 = not determined). Increasing positive or negative delta CT values are given in brighter green or red colors, respectively. Black indicates that either the gene is not expressed or is below the limit of detection.

Collagen 1 was significantly over expressed in the textured implant group on day 60 compared with both smooth implant groups on days 60 and 120 (RQ day 60 smooth vs. day 60 textured: 0.11±1.04, p<0.05; RQ day 120 smooth vs. day 60 textured: 0.16±0.95, p<0.05). Significant differences in the expression of collagen types 2, 3 and 4 were not detected among study groups. However, Collagen 2 trended towards highest expression in the smooth implant group on day 60 and decreased over the study period, that is, from day 60 to day 120 (Fig 14). In general, collagen subtypes appeared to be expressed less in smooth implant groups. With respect to inflammatory markers, significant differences were only detected for CD68; namely, there was lower expression in the smooth implant group on day 120 compared with the textured implant group on day 60 (RQ day 60 textured vs. day 120 smooth: 1.96±0.17; p<0.05) (Fig 15). Significant differences among study groups were not detected in any of the other investigated genes. Of note, inflammatory marker IL 10 was only present in the textured implant group on day 60 and not detectable anymore on day 120. In contrast, the expression of fibrotic markers, namely TGFbeta3 and 1, as well as Smad3 remained stable over the study period and unrelated to the implant type. Results are given in Fig 16 as heat map with increasing positive or negative delta CT values in brighter green or red colors, respectively. Black indicates that either the gene is not expressed or is below the limit of detection.

## Discussion

This study demonstrates significant differences in *in-vivo* capsule formation between smooth and textured silicone implants. Whereas smooth implant surfaces led to thinner capsules, collagen density was significantly higher compared to textured implant surfaces. These histological findings were substantiated by *in-vivo* imaging with means of 7-Tesla MRI and high-resolution ultrasound and associated with significant differences in gene expression of pro-fibrotic and inflammatory markers. In the long-term, however, differences equalized and both textured and smooth implants revealed comparable results regarding thickness and density.

The impact of implant surface properties on capsule formation is still controversially discussed in current literature. Whereas most of the studies indicate a reduction of fibrous tissue deposition after surface texturing, some authors demonstrate no significant differences compared with smooth surfaces [11, 18, 19].

Clugston et al. investigated effects of surface texturing on capsular contracture in a rat model. Non-quantitative histological analysis revealed comparable findings within each group but with a tendency for thinner and less aligned collagen fibers after textured implants [18]. Recently, Minami et al. performed a detailed study of histologic alterations in capsule formation after smooth and textured implants in a pig model [20], where comparison of both implant types did not show any significant differences at one specific time point, but capsule thickness varied significantly at different time points within the same study group. Whereas smooth implants provoked a constant increase in capsule thickness over the 270 days study period, textured surfaces led to a peak on day 180 after which the capsule declined again. In our study textured implants led to a comparable peak in capsule thickness in the mid-term. Furthermore, we were able to verify this histological aspect in-vivo by HR-US and 7-Tesla MRI. Especially the latter provides a unique tool to assess the morphology of the entire fibrotic capsule by 3D reconstruction and volumetric measurements. Concomitant to thickness, the volume of the fibrotic capsule was altered with higher volumes on day 60 in the textured implant group but comparable volumes across surface groups at the later time point. Interestingly, surface areas did not vary between study dates, but they did between implant types. As contracture develops it has the effect of decreasing the surface area of the implant until the implant reaches a spherical shape, at which point further contraction is impossible because of the non-compressibility of liquids [21]. In our opinion this is the most accurate method to define contracture as it reassembles the entire capsule and thus a complete overview of the contracture process. Our results indicate a more contracted capsule due to the smooth implant surface, as surface area was lower in this group. However, none of the MRI-based results were statistically significant what was most likely associated with still insufficient resolution capacity despite latest 7-tesla MRI technology as well as low sample size (n = 2). The latter might be of higher significance as thickness values did correlate with both histology and HR-US measurements. From an economical point of view HR-US is the preferred method for thickness measurements as it is remarkable capable to identify and assess the fibrotic capsule in-vivo. Of note, thickness measurements in MRI were thicker compared to both histology and HR-US, due to the fact that measurements were performed from the implant shell to the outer margin of the skin. The latter was necessary in MRI as the resolution in some samples was insufficient for proper identification of the sole capsule.

With respect to collagen density, several studies demonstrated loose collagen structures related to textured surfaces. Smahel et al. revealed less compact capsule development 8 months after insertion of textured surfaces in rats [22]. In a clinical study of Wyatt et al. smooth implants more often had a dense collagenous capsule than textured implants after 5 years of follow-up [23]. Furthermore, the incidence of capsules with collagen fibers arranged parallel to the implant surface was significantly greater in the smooth group compared to the textured group. Our results are in accordance to these findings as capsules of textured implants were significantly less dense than those of smooth implants. Thus surface texturing led to thicker but less dense fibrotic capsules, leading to the question if total collagen deposition differs among both implant types. Bucky et al. compared smooth, textured and polyurethane covered implants in rabbits and found lowest Collagen 3 deposition around textured surfaces [24]. Minami et al. showed that over time thin collagen fibers were replaced by thick fibers independent of the implant type [20]. Thereby thin and thick fibers equate to collagen types 3 and 1, respectively [25]. In our study, analysis of gene expression revealed a significant increase of Collagen 1 as well as a remarkable trend of Collagen 3 towards over expression both related to textured implants in the mid-term. However, despite mid-term up-regulation of collagen gene expression, collagen deposition revealed no significant differences in the long term.

As fibrosis is evidentially related to inflammation, Meza-Brites et al. investigated periprosthetic breast capsules and immunophenotypes of inflammatory cells in 80 breast implant capsules and found textured implants to induce a stronger local T-cell immune response [26, 27]. In our study, gene expression of CD68, a macrophage marker, was slightly increased on day 60 in the textured implant group when compared to the smooth group, and IL10 was detectable textured but not in smooth surfaces. Interestingly, CD68 gene expression was significantly higher in the smooth implant group on day 120. Although this may indicate an increase of inflammation in the long run, significant upregulation of other inflammatory markers such as IL4 and IL13 was not observed at any time point. An alternative explanation was highlighted in recent studies, which found that especially in skin related tissues, CD68 is not macrophage-specific but also expressed by fibroblasts [27, 28]. Therefore, our findings may indicate an increase of fibroblast proliferation and thus stronger fibrotic reaction associated with smooth implant surfaces in the long term. However, no significant differences related to time or implant surface topography were observed for the expression of remaining fibrotic markers TGFbeta1 and 3, as well as Smad3.

With regard to the clinical translation of our findings, several studies investigated on histological criteria and their correlation to the clinical appearance of capsular fibrosis.

Whereas most of the data in current literature indicate that capsule thickness correlates significantly with occurrence of capsular contracture, interestingly, none of these studies assessed the density of the fibrotic capsules [29–31]. Furthermore, there is contrary evidence that thickness of the capsule has no impact on clinical appearance, and does not correlate at all [32, 33]. In a recently published study about histological findings in capsule tissue specimens, Bui et al. assessed thickness and density and demonstrated that both criteria significantly correlate with symptoms [31]. In accordance to these findings Rubino et al. demonstrated via electron microscopy that contracted capsules of textured implants were not only thicker but also more compact compared to uncontracted capsules [34]. Of note, contracted capsules from textured implants still obtained a layer of irregular and woven appearance, whereas contracted capsules from smooth implants showed dense fibers throughout the entire capsule despite similar thickness. The latter highlights the impact of capsule density and that sole thickness of the capsule is insufficient for translation into clinical contracture. From a histomorphological point of view, contracture would rather come with an increase in density than thickness, which is even more likely to decrease due to the contracture process itself.

Therefore, our data indicates that textured implants are less prone to capsular contracture in the mid-term but comparable to smooth surfaces in the long-term. Furthermore, our results provide the histomorphological correlate to clinical studies demonstrating a delayed occurrence of capsular contracture after textured implants [35, 36] as well as to those that state an absolute decrease of capsular contracture incidence irrespective of the follow-up period [13].

Another important aspect that needs to be addressed in the context of surface properties and capsule formation is the occurrence of implant-associated anaplastic large cell lymphoma (ALCL). Although the U.S. Food and Drug Administration stated that ALCL should not be of major concern to patients considering silicone implants as the absolute risk remains very low, more and more cases recently appeared in current literature [37]. Interestingly, according to Brody et al. all silicone implant associated ALCL reported thus far were diagnosed in patients, who had at least once in their past medical history an implant with a textured surface [38]. Although we did not find any clinical or histological signs for ALCL in this study (data not shown), the impact of this finding should not be overestimated. In contrast to the clinical conjecture of rough surfaces as a cause for implant associated ALCL, Oppenheimer found that smooth surfaced foreign bodies implanted in animals were more prone to sarcoma formation, whereas rough or irregular surfaces did not generate tumors [39]. Further studies confirm Oppenheimer’s finding in laboratory animals and thus raising questions about the relevance of animal experiments for implant associated ALCL in general [40, 41].

There are several limitations to our study. Although considered as major contributor to the fibrotic process after silicone implant insertion, biofilm formation was not evaluated in this study. With respect to current literature, biofilm formation is only detectable in animal models if implant pockets were inoculated with bacteria previous to implant insertion [8, 42–44]. Thereby, Jacombs et al. could demonstrate that biofilm and capsule formation after smooth and textured implants revealed no significant differences among study groups and concluded that once a threshold of biofilm occurs, irrespective from surface properties, there seems to be an equal propensity to progress to capsular contracture [43]. These findings were substantiated by Pajkos et al., who demonstrated in a clinical study that biofilm formation was significantly associated with capsular contracture but its occurrence did not correlate with smooth or textured implant surfaces [45]. Therefore we decided to exclude biofilm formation as outcome measurement as it is evidentially not related to surface properties relevant for capsule formation. Furthermore, we wanted to prevent bacteria inoculation, which would provide significant bias rather than highlight the impact of implant surfaces on capsule formation. From a clinical point of view, capsular contracture is defined by its symptoms, namely palpable hardness, visible deformities or pain, of which the latter is an absolute indication for surgical intervention. Therefore, capsule thickness, collagen density as well as gene expression of fibrotic or inflammatory markers cannot mimic clinical symptoms and thus remain without therapeutic consequences. Nevertheless, pain is difficult to evaluate, even in humans, and applanation tonometry or biochemical analysis of tissue compliance correlated well with histological findings in former studies [18, 20, 24]. Furthermore, macroscopic, histological and biochemical alterations in our study can explain the vast range of incidence of capsule contracture reported in clinical studies, which is apparently not only related to the implant surface but also to the time point of investigation [5].

## Conclusion

Silicone implants with textured surfaces lead to temporarily thicker but less dense fibrotic capsules compared to smooth surfaces. This difference was apparent in histology, MRI and high-resolution ultrasound and substantiated by fibrotic and inflammatory gene expression. Differences, however, equalized over time, revealing comparable results in the long-term and thus highlighting the impact of time on capsule evaluation. In addition, MR-based measurements provide unique options to quantify capsular fibrosis development and to objectify the occurrence of contracture.
